# Editorial: Lipid-protein interactions and regulation in neurodegenerative disorders

**DOI:** 10.3389/fnmol.2023.1167046

**Published:** 2023-03-14

**Authors:** Mohammad Haddadi, Mehrnaz Narooei-Nejad

**Affiliations:** ^1^Department of Biology, Faculty of Basic Sciences, University of Zabol, Zabol, Iran; ^2^Department of Medical Genetics, School of Medicine, Zahedan University of Medical Sciences, Zahedan, Iran

**Keywords:** lipid-protein interaction, neurodegenerative diseases, protein aggregation, lipid vesicles, lipid species, Electroacupuncture, prion protein, amyloid beta

Lipids in general are the most abundant organic molecules in the central nervous system (CNS). Each lipid species implements a specific function mainly as a platform for other molecular players including proteins. Lipid molecules can act as transmitters to convey signals in intra- or inter-cellular contexts. Moreover, lipids can impose structural alteration on misfolded proteins and impact their neurotoxicity. With such a broad functional capacity, the role of lipids in neurodegenerative conditions is essential to understanding disease mechanisms and developing an effective therapeutic intervention.

The main aim of this Research Topic was to provoke research interest in studying lipids as dynamic and active macromolecules in the context of neuronal function. This Research Topic brings together three review papers on lipid-protein interactions, Aβ structure at the atomic level, and conversion of prion protein to its toxic form as well as two original research articles introducing novel, innovative, and applicable methods to investigate lipid-lipid and lipid-protein interactions and demonstrate an ameliorative intervention to reduce damaging effects of post-traumatic stress disorder (PTSD).

Andersson et al. have developed a fluorescence microscopy assay to identify and study monomeric protein-lipid interaction and lipid exchange between vesicular membranes at a single event level and also the frequency of occurrence of each event. A MATLAB program has been developed and introduced to overcome imperfections during image processing. This technique employs red and blue channels to study colocalization and intensity changes of lipid vesicles in the presence of proteins. Based on known interaction between monomeric α-syn and acidic lipid molecules such as monosialotetrahexosylganglioside, the team has shown that the α-syn can enhance vesicle fission and disrupt vesicle trafficking in the neuron termini. Although they used unlabeled protein, this method has the capacity to analyze the interaction between fluorescently labeled protein molecules and lipid vesicles in terms of the absorbed amount of protein per vesicle. and to investigate fission, fusion, and lipid exchange events, as well. Altogether, this microscopy assay offers an excellent method to conduct protein-lipid interactive studies, though it can still be improved by others for further fruitful technical achievements.

Zhou et al. investigated the neuroprotective effect of Electroacupuncture (EA) treatment in altering the lipid composition of mice brain tissue following post-traumatic stress disorder (PTSD). Acupuncture is a traditional Chinese medicine with the capacity to ameliorate learning and memory shortcomings and emotion dysregulation. PTSD is a behavioral state following a traumatic event experience. In general, about 10% of the world's population is suffering from PTSD, a condition which was raised in prevalence by and COVID-19 pandemic. Considering the role of lipids in the development of some psychiatric disorders, PTSD-related changes in brain lipidome remain elusive. Zhou et al. reported the changes in brain lipid content soon after experiencing traumatic stress and the neuromodulatory action of EA, for the first time. They applied a modified stress procedure in which first the animals were restrained and then forced to swim and inhale ether until complete consciousness and finally received a foot electric shock. This protocol named modified single prolonged stress (mSPS) can cause some sort of anxiety and fear of learning defects. Zhou et al. showed that mSPS cause remarkable changes in the hippocampus and prefrontal cortex lipid composition, mainly sphingolipids, glycerolipids, and fatty acyls. Early interventions mediated by EA showed promises for ameliorating mSPS-induced PTSD-like behaviors and lipid changes *via* regulating brain lipidome to the normal status.

Vendruscolo has discussed the necessity of lipid homeostasis for the normal function of living organisms. In this review, it has been illustrated that changes in the lipid homeostasis system result in cellular lipidome remodeling, which can trigger the formation of toxic lipid species. These toxic lipids contribute to protein condensation and aggregation. Considerable data are compiled to demonstrate the thermodynamic characteristics of proteins in the cell, highlighting the importance of protein homeostasis system in preventing protein condensation and protein conversion to dysfunctional peptides. The connection between the protein homeostasis system and other cellular components, mainly lipids, is discussed very well to address dysregulated protein condensation as an outcome of unbalanced lipid homeostasis.

Yang et al. presented new findings on Aβ structure at the atomic level, helping scientists to realize Aβ behavior as a pathogenic agent affecting the brain and other peripheral organs. As the Aβ hypothesis is the most prominent accepted theory for Alzheimer's disease etiology, more structural information on Aβ structure will lead to a better understanding of its function, finding reliable biomarkers for AD diagnosis and offering more efficient therapeutic strategies. Though there is no straightforward discussion on Aβ-lipid interaction, the diversity of the organized contents provides extremely valuable data about Aβ poly peptide structure and function which can be the basis for further confined studies regarding lipids involvement in Aβ toxicity.

Cellular prion protein (PrPc) can get converted to its pathogenic form upon interacting with lipids. Conceição et al. have reviewed molecular events underlying transmissible spongiform encephalopathies (TSEs), shedding light on the conversion of the PrPC into a pathogenic prion protein (PrP) called prion scrapie (PrPsc), as the main causal factor of TSEs. In fact, PrPsc is prone to make amorphous and amyloid aggregates in the CNS of TSE patients. These aggregates have hydrophobic regions and may get inserted into the lipid membranes, leading to some kind of destabilization. Albeit, DNA and RNA molecules can act as cofactors for this conversion alongside lipid molecules. Localization of PrPc on the lipid raft at the outer cell membrane is mediated through glycosylphosphatidylinositol (GPI) molecules, allowing PrP interaction with various ligands and membrane lipids. Alternations in GPI, detachment of PrP, rise in cholesterol level and changes in sphingolipid and glycerophospholipid metabolism are discussed as the main events contributing to PrP to PrPsc conversion. As alterations in protein structures are hallmarks of Alzheimer's and Parkinson's disease, revealing the type and mechanism of PrP interaction with lipids seems helpful to unravel these kinds of interactions in other related disease conditions.

We hope this Research Topic attracts the attention of neuroscience scholars to lipid molecules, as highly important but less studied macromolecules and the cornerstone of neuronal function in healthy conditions. Finally, we tried to elucidate the contribution of lipid molecules in neurodegenerative disorders, as well.

## Author contributions

MH and MN-N have analyzed the submitted papers and provided a brief explanatory description for each paper. MH has written and edited the manuscript. All authors contributed to the article and approved the submitted version.

